# Risk score to predict gastrointestinal bleeding after acute ischemic stroke

**DOI:** 10.1186/1471-230X-14-130

**Published:** 2014-07-25

**Authors:** Ruijun Ji, Haipeng Shen, Yuesong Pan, Penglian Wang, Gaifen Liu, Yilong Wang, Hao Li, Aneesh B Singhal, Yongjun Wang

**Affiliations:** 1 Tiantan Comprehensive Stroke Center, Tiantan Hospital, Capital Medical University, No.6 Tiantanxili, Dongcheng District, Beijing 100050, China; 2 Department of Statistics and Operation Research, University of North Carolina, Chapel Hill, NC, USA; 3 Department of Neurology, Massachusetts General Hospital, Boston, MA, USA

## Abstract

**Background:**

Gastrointestinal bleeding (GIB) is a common and often serious complication after stroke. Although several risk factors for post-stroke GIB have been identified, no reliable or validated scoring system is currently available to predict GIB after acute stroke in routine clinical practice or clinical trials. In the present study, we aimed to develop and validate a risk model (acute ischemic stroke associated gastrointestinal bleeding score, the AIS-GIB score) to predict in-hospital GIB after acute ischemic stroke.

**Methods:**

The AIS-GIB score was developed from data in the China National Stroke Registry (CNSR). Eligible patients in the CNSR were randomly divided into derivation (60%) and internal validation (40%) cohorts. External validation was performed using data from the prospective Chinese Intracranial Atherosclerosis Study (CICAS). Independent predictors of in-hospital GIB were obtained using multivariable logistic regression in the derivation cohort, and β-coefficients were used to generate point scoring system for the AIS-GIB. The area under the receiver operating characteristic curve (AUROC) and the Hosmer-Lemeshow goodness-of-fit test were used to assess model discrimination and calibration, respectively.

**Results:**

A total of 8,820, 5,882, and 2,938 patients were enrolled in the derivation, internal validation and external validation cohorts. The overall in-hospital GIB after AIS was 2.6%, 2.3%, and 1.5% in the derivation, internal, and external validation cohort, respectively. An 18-point AIS-GIB score was developed from the set of independent predictors of GIB including age, gender, history of hypertension, hepatic cirrhosis, peptic ulcer or previous GIB, pre-stroke dependence, admission National Institutes of Health stroke scale score, Glasgow Coma Scale score and stroke subtype (Oxfordshire). The AIS-GIB score showed good discrimination in the derivation (0.79; 95% CI, 0.764-0.825), internal (0.78; 95% CI, 0.74-0.82) and external (0.76; 95% CI, 0.71-0.82) validation cohorts. The AIS-GIB score was well calibrated in the derivation (P = 0.42), internal (P = 0.45) and external (P = 0.86) validation cohorts.

**Conclusion:**

The AIS-GIB score is a valid clinical grading scale to predict in-hospital GIB after AIS. Further studies on the effect of the AIS-GIB score on reducing GIB and improving outcome after AIS are warranted.

## Background

Gastrointestinal bleeding (GIB) is a serious complication after acute stroke with an estimated incidence of 1%-5% [1-8]. Several risk factors for post-stroke GIB have been identified [2,6-9], such as advanced age, medical history of peptic ulcer or previous GIB, admission stroke severity, and impaired level of consciousness. However, no reliable or validated scoring system is currently available to predict GIB after acute stroke in routine clinical practice or clinical trials. An effective risk stratification model would be helpful to identify vulnerable patients, allocate relevant medical resources, and contrapuntally implement prophylactic strategies, such as the use of histamine H_2_ receptor antagonists (H_2_RAs) or proton pump inhibitors (PPIs) [10-18]. A predictive scoring system would also be useful in clinical trials and health outcomes research by providing an objective method to risk-adjust when determining endpoints. In the present study, we aimed to develop and validate a risk score (Acute Ischemic Stroke associated Gastrointestinal Bleeding Score, AIS-GIB score) for predicting GIB during acute hospitalization after acute ischemic stroke (AIS).

## Methods

### Derivation, internal and external validation cohorts

The derivation and internal validation cohorts were obtained from the largest stroke registry in China, the China National Stroke Registry (CNSR), which is a nationwide, multicenter, prospective registry of consecutive patients with acute cerebrovascular events [19]. Briefly, hospitals in China are classified into 3 grades: I (community hospitals); II (hospitals that serve several communities); or III (central hospitals for a certain district or city). The CNSR includes 132 hospitals including 100 grade III and 32 grade II hospitals covering 27 provinces and 4 municipalities across China. These sites were carefully selected from a total of 242 urban and rural hospitals by the CNSR steering committee based on their research capability and commitment to the registry. Trained research coordinators at each hospital review medical records daily to screen, consent and enroll consecutive patients. To be eligible for this study, subjects had to meet the following criteria: (1) age 18 years or older; (2) hospitalized with a primary diagnosis of AIS according to the World Health Organization (WHO) criteria [20]; (3) stroke confirmed by head computerized tomography (CT) or brain magnetic resonance imaging (MRI); (4) direct admission to hospital from a physician’s clinic or emergency department. Eligible patients from the CNSR were randomly divided into derivation (60%) and validation (40%) cohorts.

The external validation cohort was derived from the Chinese Intracranial Atherosclerosis Study (CICAS) [21], which was a hospital-based, multicenter, prospective study aiming at investigating the incidence, risk factors and impact of intracranial atherosclerosis among patients with AIS. Inclusion criteria of the CICAS were: (1) age 18 to 80 years; (2) symptom onset within 7 days; (3) hospitalized with a primary diagnosis of AIS or transient ischemic attack (TIA). Exclusion criteria of the CICAS were: (1) pre-admission modified Rankin Scale (mRS) score ≥ 3; (2) inability to undergo MRI for cerebral vascular imaging. For the present study, patients diagnosed with TIA were excluded.

Informed consent was obtained for all patients enrolled in the CNSR and CICAS. The scientific use of data registered in the CNSR and CICAS was approved by the central institutional review board at Beijing Tiantan Hospital and local ethical committees at each participating hospital in the CNSR and CICAS (Additional file 1: Appendix A and B).

### Data collection and variables definitions

In these registries, a standardized case report form (CRF) is used for data collection. Relevant data is extracted from medical records by trained research coordinators. Data from each CRF is manually checked for completeness, correct coding, and proper application of diagnostic algorithms by research specialists from an independent contract research organization.

In the present study, the following candidate variables were analyzed: (1) demographics (age and gender); (2) stroke risk factors: hypertension (history of hypertension or anti-hypertensive medication use), diabetes mellitus (history of diabetes mellitus or anti-diabetic medication use), dyslipidemia (history of dyslipidemia or lipid-lowering medication use), atrial fibrillation (history of atrial fibrillation or documentation of atrial fibrillation on admission), coronary heart disease, peripheral artery disease, history of stroke/TIA, current smoking, and excess alcohol consumption (≥2 standard alcohol beverages per day); (3) preexisting comorbidities: congestive heart failure, valvular heart disease, chronic obstructive pulmonary disease (COPD), hepatic cirrhosis, peptic ulcer, previous GIB, renal failure, arthritis, Alzheimer’s disease/dementia, and cancer; (4) pre-stroke functional status based on the mRS score derived from the medical record, categorized as mRS < 3 or ≥ 3; (5) pre-admission anticoagulant (warfarin) use or anti-platelet medication use (aspirin, clopidogrel, or extended release dipyridamole combined with aspirin); (6) admission systolic and diastolic blood pressure (mm Hg); (7) admission stroke severity based on the National Institutes of Health Stroke Scale score (NIHSS) score and the Glasgow Coma Scale (GCS) score; (8) stroke subtype according to the Oxfordshire Community Stroke Project (OCSP) criteria [22] where AIS is classified into partial anterior circulation infarct (PACI), total anterior circulation infarct (TACI), lacunar infarction (LACI), and posterior circulation infarct (POCI); (9) intravenous tissue plasminogen activator (t-PA) thrombolysis within 3 h after onset; (10) antiplatelet or antithrombotic therapy on admission; (11) length of hospital stay (days).

GIB was defined according to Davenport et al. [2] as any episode of fresh blood or coffee ground emesis, hematemesis, melena, or hematochezia occurring during index hospitalization. GIB after AIS were diagnosed by treating physicians (with or without blood transfusion) and prospectively registered by trained research coordinators. Only GIB developed during hospitalization was documented and GIB occurred before admission was not considered. Data on the development of GIB after AIS were manually checked for completeness, correct coding, and proper application of diagnostic algorithm by a research specialist from an independent contract research organization.

### Statistical analysis

Model building was performed exclusively in the derivation cohort. In univariate analysis, Chi-square and Mann-Whitney tests were used as appropriate. Multivariable logistic regression was used to determine independent predictors for GIB after AIS in the derivation cohort. Candidate variables were those with biologically plausible link to GIB on the basis of prior publication and those associated with GIB in univariate analysis (P < 0.1). On multivariable analysis, backward stepwise method was used. To test for collinearity between the covariates of the final multivariable model, the tolerance and variance inflation factor (VIF) of each covariate was calculated. The β-coefficients from the final model were used to generate a point scoring system of the AIS-GIB score, as in previous studies [23]. The resulting risk score was then validated by assessing model discrimination and calibration in the internal and external validation cohort [24]. Discrimination, i.e. the degree to which the prognostic score enables the discrimination between patients with and without GIB after AIS, was assessed by calculating the area under the receiver operating characteristic curve (AUROC). An AUROC of 1.0 indicates perfect prediction, whereas a C statistic of 0.5 indicates no better than random prediction. Calibration, i.e. the agreement between predicted and observed risk of GIB, was assessed by performing the Hosmer-Lemeshow goodness-of-fit test and was graphically depicted in the plot of observed versus predicted GIB risk according to 10 deciles of predicted risk.

All tests were 2-tailed and statistical significance was set at a value of p < 0.05. Statistical analysis was performed using SAS 9.1 (SAS Institute, Cary, NC) and SPSS 17.0 (SPSS Inc., Chicago, IL).

## Results

### Patient characteristics

Patient characteristics in the derivation, internal and external validation cohorts were shown in Table 1. From September 2007 to August 2008, a total of 14,702 patients with AIS were enrolled in the CNSR. The median age was 66 years (IQR 58-75) and 62% were male. The median length of hospital stay (LOS) was 14 days (IQR, 10-21). The median admission NIHSS score was 5 (IQR, 2-9). A total of 362 (2.5%) patients had GIB during hospitalization, of whom 39 (0.3%) required blood transfusion. The derivation cohort (n = 8,820) and internal validation cohort (n = 5,882) were matched with respect to baseline characteristics and rates of in-hospital GIB (Table 1).

**Table 1:** Patient characteristics

	**Derivation cohort**	**Internal validation cohort**	**P**_**1**_value*	**External validation cohort**	**P**_**2**_value*
	**(n = 8,820)**	**(n = 5,882)**		**(n = 2,938)**	
Demographics					
Age, y, median (IQR)	66 (56-74)	66 (57-75)	0.11	64 (55-72)	<0.001
Gender (male), n (%)	5430 (61.6)	3675 (62.5)	0.27	1927 (65.5)	<0.001
Vascular risk factor, n (%)					
Hypertension	5601 (63.5)	3683 (62.6)	0.27	1987 (67.6)	<0.001
Diabetes mellitus	1834 (20.8)	1287 (21.9)	0.11	720 (24.5)	<0.001
Dyslipidemia	947 (10.7)	637 (10.8)	0.86	386 (13.1)	<0.001
Atrial fibrillation	643 (7.3)	415 (7.1)	0.60	175 (6.0)	0.01
Coronary artery disease	1222 (13.9)	811 (13.8)	0.91	285 (9.7)	<0.001
Peripheral artery disease	64 (0.7)	29 (0.5)	0.08	26 (0.9)	0.39
History of stroke/TIA	2795 (31.7)	1822 (31.0)	0.36	809 (27.5)	<0.001
Smoking	3510 (39.8)	2326 (39.5)	0.70	1022 (34.8)	<0.001
Heavy alcohol consumption	1346 (15.3)	921 (15.7)	0.55	372 (12.7)	<0.001
Others coexistent condition, n (%)					
Congestive heart failure	169 (1.9)	121 (2.1)	0.55	24 (0.8)	<0.001
Valvular heart disease	213 (2.4)	139 (2.4)	0.83	40 (1.4)	0.001
COPD	98 (1.1)	64 (1.1)	0.88	12 (0.4)	0.001
Hepatic cirrhosis	29 (0.3)	21 (0.4)	0.78	7 (0.2)	0.44
Peptic ulcer or previous GIB	283 (3.2)	195 (3.3)	0.72	76 (2.6)	0.09
Renal failure	7 (0.1)	4 (0.1)	0.80	3 (0.1)	0.71
Arthritis	266 (3.0)	176 (3.0)	0.27	45 (1.5)	<0.001
Dementia	113 (1.3)	82 (1.4)	0.57	18 (0.6)	0.003
Cancer	150 (1.7)	109 (1.9)	0.51	54 (1.8)	0.62
Pre-stroke dependence (mRS ≥3), n (%)	809 (9.2)	535 (9.1)	0.87	0 (0.0)	<0.001
Pre-admission antiplatelet therapy, n (%)	1449 (16.4)	932 (15.8)	0.35	357 (12.2)	<0.001
Pre-admission anticoagulation therapy, n (%)	210 (2.4)	122 (2.1)	0.22	26 (0.9)	<0.001
Admission NIHSS score, median (IQR)	5 (2-9)	5 (2-9)	0.68	4 (2-8)	0.02
Admission GCS score, median (IQR)	15 (14-15)	15 (14-15)	0.36	15 (15-15)	0.18
Admission SBP (mmHg), median (IQR)	150 (134-163)	150 (135-162)	0.88	150 (135-167)	0.22
Admission DBP (mmHg), median (IQR)	89 (80-95)	89 (80-95)	0.98	90 (80-98)	0.11
OCSP subtype, n (%)			0.22		<0.001
Partial anterior circulation infarct (PACI)	4834 (54.8)	3327 (56.6)		1829 (62.3)	
Total anterior circulation infarct (TACI)	811 (9.2)	519 (8.8)		176 (6.0)	
Lacunar infarction (LACI)	1667 (18.9)	1074 (18.3)		246 (8.4)	
Posterior circulation infarct (POCI)	1508 (17.1)	962 (18.4)		687 (23.4)	
Intravenous t-PA within 3 h after onset, n (%)	108 (1.2)	73 (1.2)	0.93	137 (4.6)	<0.001
Antithrombotic therapy on admission, n (%)	7371 (83.6)	4950 (84.2)	0.35	2550 (86.8)	<0.001
Anticoagulation therapy on admission, n (%)	210 (2.4)	122 (2.1)	0.24	159 (5.4)	<0.001
Length of hospital stay (days), median (IQR)	14 (10-20)	14 (10-20)	0.96	14 (11-18)	0.15
In-hospital GIB, n (%)	227 (2.6)	135 (2.3)	0.29	44 (1.5)	0.001

*P_1_ denotes the P value comparing the derivation and internal validation cohorts; P_2_ denotes the P value comparing the derivation and external validation cohorts.

*Abbreviations*: *IQR* interquartile range, *COPD* chronic obstructive pulmonary disease, *mRS* modified Rankin Scale, *NIHSS* National Institutes of Health Stroke Scale score, *GCS* Glasgow Coma Scale, *SBP* Systolic Blood Pressure, *DBP* Diastolic Blood Pressure, *OCSP* Oxfordshire Community Stroke Project, *t-PA* tissue plasminogen activator.

From October 2007 to June 2009, a total of 3,580 patients were registered in 22 participating hospitals in the CICAS network. Of these, we excluded 329 patients with TIA (9.1%) and an additional 313 patients (8.7%) who were missing data for one or more covariates used in the AIS-GIB score. The final external validation cohort comprised 2,938 patients with AIS (Table 1). The median LOS was 14 days (IQR, 11-18). The median admission NIHSS score was 4 (IQR, 2-8). A total of 44 (1.5%) patients had GIB during hospitalization, of whom 7 (0.2%) required blood transfusion.

### Predictors of GIB and derivation of the AIS-GIB score

The univariate analysis for potential predictors for GIB after AIS in the derivation cohort is shown in Additional file 1: Table S1. The results of the multivariate analysis, including β-coefficients for each independent predictor, are shown in Table 2. The tolerance of covariates in the final multivariable model ranged between 0.68 and 0.99; the mean VIF was 1.28 (range: 1.01-1.75).

**Table 2:** Multivariable predictors of GIB after AIS from the derivation cohort (n = 8,820)

**Variables**	**β-coefficients**	adjusted O.R.*	**95% C.I.**	**P value**
Model intercept	-6.529			
Age (years)	0.009	1.01	1.00-1.02	0.01
Gender (male)	0.370	1.45	1.13-1.85	0.004
Hypertension	0.345	1.41	1.10-1.82	0.008
Hepatic cirrhosis	1.299	3.66	1.17-11.5	<0.001
Peptic ulcer or previous GIB	1.226	3.41	2.28-5.10	<0.001
Pre-stroke dependence (mRS ≥3)	0.717	2.05	1.54-2.72	<0.001
Admission NIHSS (per 1 point increase)	0.063	1.07	1.04-1.09	<0.001
Admission GCS (per 1 point decrease)	0.057	1.06	1.01-1.11	0.01
OCSP subtype				
Lacunar infarction (LACI)	…	1.00	…	…
Partial anterior circulation infarct (PACI)	0.169	1.18	0.80-1.75	0.39
Total anterior circulation infarct (TACI)	0.457	1.58	1.01-2.49	0.02
Posterior circulation infarct (POCI)	0.804	2.24	1.47-3.40	<0.001

*Multivariable logistic regression adjusted for age, gender, stroke risk factors, comorbidities, pre-stroke dependence, pre-admission antiplatelet therapy, pre-admission anticoagulation therapy, admission blood pressure, NIHSS score, GCS score, OCSP subtypes, intravenous t-PA, antithrombotic therapies within 48 h after admission, and length of hospital stay.

*Abbreviation*: *GIB* gastrointestinal bleeding, *AIS* acute ischemic stroke, *OR* odds ratio, *C.I.* confidence interval, *mRS* modified Rankin Scale, *NIHSS* National Institutes of Health Stroke Scale score, *GCS* Glasgow Coma Scale, *OCSP* Oxfordshire Community Stroke Project, *t-PA* tissue plasminogen activator.

The β-coefficients from the multivariable regression model were used to generate scoring system of the AIS-GIB score (Table 3). To derive an integer value for each predictor, the β-coefficient of patients younger than 60 years was used as reference and the value was rounded to the closest integer. When we used β-coefficient of different variables in the multivariable model as reference, such as female gender, patients without medical history of hypertension, patients with admission NIHSS score ≤4, or patients of lacunar infarction, although the scoring system was different, the discrimination of the final model was similar (data not shown). The probability of GIB can be estimated for an individual patient by summing points assigned to each predictor to create a total point score that ranges from 0 to 18. The median AIS-GIB score was 3 (IQR: 2-5; range: 0-15) in the derivation cohort.

**Table 3:** The point scoring system of the AIS-GIB score

**Items**	**Score**	**Point total**	Estimate of risk (%)*
**Age group**		0	0.3
≤ 59	0	1	0.4
60-69	1	2	0.6
70-79	1	3	1.0
≥80	2	4	1.5
**Gender**		5	2.4
Female	0	6	3.7
Male	1	7	5.7
**Medical history**		8	8.7
Hypertension	1	9	13.0
Hepatic cirrhosis	3	10	19.0
Peptic ulcer or previous GIB	3	11	26.8
**Pre-stroke disability (mRS** ≥ **3)**	2	12	36.5
**Admission NIHSS score**		13	47.4
0-4	0	14	58.6
5-9	1	15	68.9
10-14	2	16	77.7
≥15	3	17	84.5
**Admission GCS score**		18	89.5
15	0		
13-14	0		
9-12	1		
3-8	1		
**OCSP subtype**			
Lacunar infarction (LACI)	0		
Partial anterior circulation infarct (PACI)	0		
Total anterior circulation infarct (TACI)	1		
Posterior circulation infarct (POCI)	2		

*The total point score gives the predicted risk of GIB after AIS according to the following Equation: Risk of GIB after AIS = 1/(1 + EXP [5.953 ‒ 0.45(Total AIS ‒ GIB score)]).

### Internal validation of the AIS-GIB score

**Figure 1:**
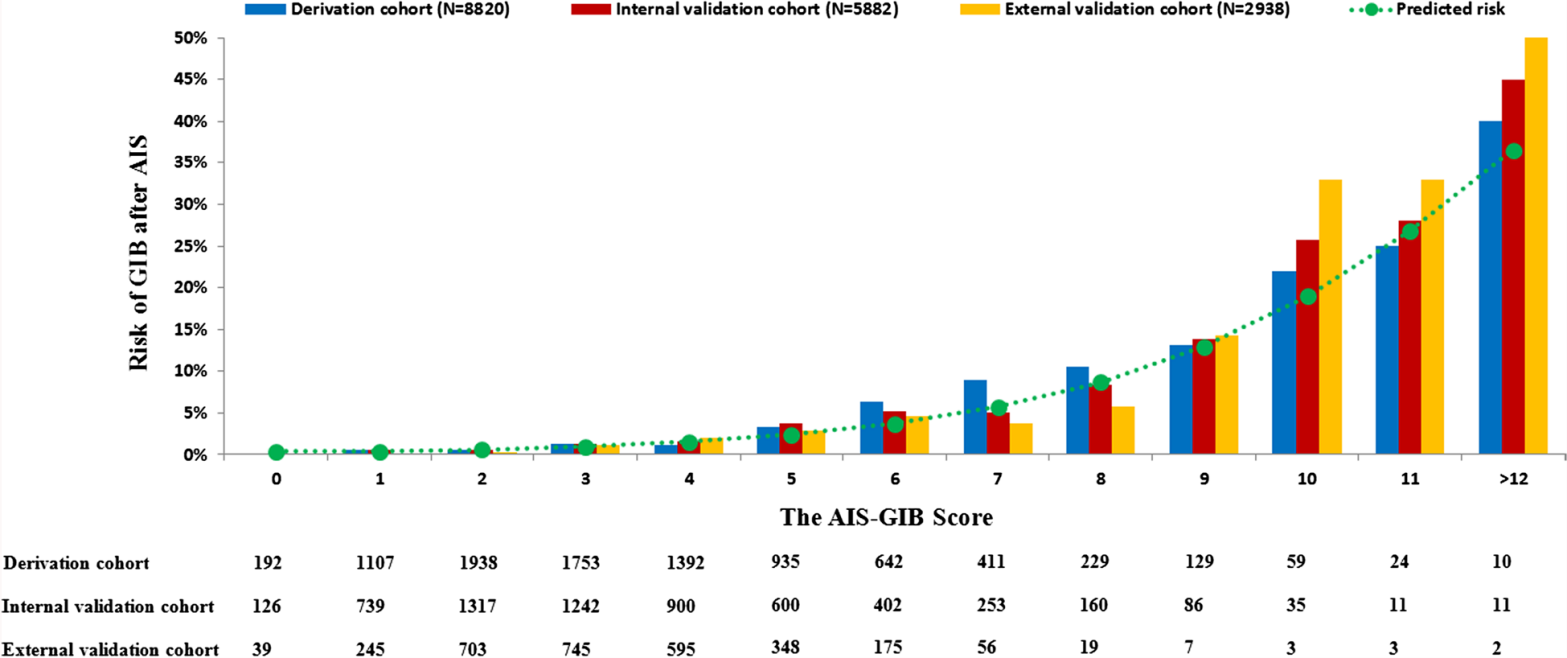
**The figure showed the proportion of in-hospital GIB after AIS according to the AIS-GIB score in the derivation (blue bar), internal (red bar) and external (yellow bar) validation cohort.** The risk of in-hospital GIB after AIS increased steadily with higher AIS-GIB score. Meanwhile, the predicted (green dot line) and observed risk of in-hospital GIB after AIS were in close agreement in the derivation, internal and external validation cohort.

Figure 1 shows the proportion of GIB after AIS according to the AIS-GIB score. The potential risk of in-hospital GIB increased steadily with higher AIS-GIB score. The discrimination of the AIS-GIB score (AUROC) with regard to any GIB in the derivation and internal validation cohort was 0.794 (95% C.I. 0.764-0.825) and 0.780 (95% C.I. 0.740-0.820), respectively. The discrimination of the AIS-GIB score for GIB requiring blood transfusion in the derivation and internal validation cohort was 0.768 (95% C.I. 0.705-0.872) and 0.771 (95% C.I. 0.712-0.923), respectively. The Hosmer-Lemeshow test was not significant (p = 0.45), and the predicted and observed risk of GIB were in close agreement according to 10 deciles of predicted risk in the internal validation cohort (r = 0.96, P < 0.001) (Additional file 1: Figure S1).

### External validation of AIS-GIB score

The AIS-GIB score showed good discrimination for any GIB (AUROC 0.755, 95% C.I. 0.710-0.818) and GIB requiring blood transfusion (AUROC 0.738, 95% C.I. 0.701-0.833) in the external validation cohort. The AIS-GIB score appeared well calibrated in the external validation cohort since the Hosmer-Lemeshow test result was not significant (p = 0.86). The plots of observed versus predicted risk of GIB showed a high correlation between observed and predicted risk according to 10 deciles of predicted risk in the external validation cohort (r = 0.91, P < 0.001) (Additional file 1: Figure S1).

### Sensitivity analysis

We performed pre-specified subgroup analyses by age, gender, and LOS. Similar good discrimination of the AIS-GIB score was seen in these subgroups (AUROC ranges 0.713-0.816, Table 4).

**Table 4:** Sensitivity analysis of the AIS-GIB in the derivation and two validation cohorts

	**Derivation cohort**		**Internal validation cohort**		**External validation cohort**	
	**(n = 8,820)**		**(n = 5,882)**		**(n = 2,938)**	
	**AUROC**	**95% C.I.**	**AUROC**	**95% C.I.**	**AUROC**	**95% C.I.**
Overall cohort	0.794	0.764-0.825	0.780	0.740-0.820	0.755	0.710-0.818
Subgroups						
Age						
≤60	0.800	0.747-0.854	0.778	0.707-0.848	0.816	0.734-0.898
≥61	0.777	0.738-0.815	0.771	0.720-0.823	0.737	0.698-0.789
Gender						
Male	0.797	0.757-0.837	0.795	0.748-0.842	0.756	0.701-0.829
Female	0.795	0.749-0.841	0.748	0.702-0.819	0.740	0.698-0.845
Length of hospital stay						
<7 days	0.786	0.730-0.841	0.792	0.708-0.876	0.713	0.639-0.786
7-14 days	0.814	0.739-0.890	0.787	0.715-0.880	0.731	0.679-0.843
>14 days	0.770	0.728-0.812	0.756	0.702-0.809	0.725	0.699-0.856

*Abbreviations*: *AUROC* area under the receiver operating characteristic curve, *C.I.* confidence interval.

## Discussion

In the present study, we derived and validated a clinical risk score that can be applied at the bedside, upon patient admission, to predict in-hospital GIB after AIS. Age, gender, certain pre-existing conditions (hypertension, hepatic cirrhosis, peptic ulcer disease, previous GIB), pre-stroke dependence, admission NIHSS score, GCS score, and OCSP subtype proved to be independent predictors for in-hospital GIB. These variables were used to develop the AIS-GIB score, which showed good discrimination and calibration in large derivation (n = 8,820), internal (n = 5,882) and external (n = 2,938) validation cohorts. In sensitive analysis, the AIS-GIB demonstrated to be valid and significant for patients with different age, gender, and length of hospital stay.

To preserve the clinical utility of the risk model for decision-making during acute hospitalization, we used only patient characteristics available at presentation or shortly after admission. This model therefore predicts the expected in-hospital GIB at presentation, and as such, the predictions could be helpful for identifying vulnerable patients, allocating relevant medical resources, implementing contrapuntal prophylactic strategies, such as the use of histamine H_2_ receptor antagonists (H_2_RAs) or proton pump inhibitors (PPIs) during subsequent hospitalization.

Several risk factors have been identified for the development GIB after acute stroke. Consistent with prior studies, we confirmed that advanced age [2,8,9], history of peptic ulcer or previous GIB [6,7,25], history of hypertension [4,26], pre-stroke dependence [2], admission stroke severity [4,6,7], impaired consciousness (measured with GCS score) [2,5,8], and middle cerebral artery territory ischemia [2,9] were significantly associated with GIB after AIS. The association between gender and GIB after acute vascular events has been controversial [25,27]. In accordance with George et al. [27], we showed that male gender was associated with the increased risk of GIB after AIS in our Chinese population. Additionally, we identified hepatic cirrhosis and posterior circulation stroke (POCI) as risk factors for developing GIB after AIS. The association between hepatic cirrhosis and increased risk of GIB after AIS might be mediated by esophagogastric varices secondary to portal hypertension or coagulopathy secondary to hepatic dysfunction. The pathophysiological mechanism underlying the association between posterior circulation ischemia and GIB is not clear. Prior studies indicated that stress ulcer may develop from what is believed to be vagal hyperactivity, which would result in increased gastric acid and pepsin secretion and damage of gastrointestinal mucosa [28,29]. Meanwhile, hyperactivity of sympathetic system after AIS tends to induce excessive catecholamine discharge, and the following vasoconstriction may result in splanchnic hypoperfusion and mucosal ischemia [29]. The association between posterior circulation ischemia and GIB might be attributed to the interruption of the autonomic nervous system pathway descending from the hypothalamus via the mesencephalon, pons, and medulla to the spinal cord. We found no association between intravenous t-PA therapy and GIB in our study, although the number of patients receiving IV t-PA in the CNSR was relatively small.

For a clinical grading scale to become widely used, it must be reliable, accurate, and practical. First, for reliability, the AIS-GIB score was developed based on a large stroke registry which included consecutive AIS patients; being a registry and not a clinical trial the data was more reflective of real-world clinical practice. Second, for accuracy, the AIS-GIB score showed good discrimination and calibration, and the results were verified in independent derivation, internal and external validation cohorts. Additionally, by sensitivity analysis, the AIS-GIB score was effective for patients with different age, gender, and LOS. Third, for convenience, the AIS-GIB score consists of factors that are readily available at hospital presentation or shortly after admission. With the scoring system showing estimated risk of in-hospital GIB after AIS for each AIS-GIB score (Table 3), clinicians can quantitatively predict the potential risk at the bedside without doing sophisticated calculation.

Prior studies have demonstrated that GIB is an important cause of morbidity and mortality in patients with acute vascular events [6,27]. In this study, one clinical question not answered is the potential effect of the AIS-GIB score on stroke outcomes. The prophylactic use of H_2_RAs, PPIs, or sucralfate is all effective in reducing GIB in critically ill patients [16-18]; however, optimum GIB prevention strategies are still unclear for patients with AIS. The AIS-GIB score could be used to identify patients at particularly high risk and then implement more aggressive monitoring (e.g. daily stool guaic testing) or more aggressive prophylaxis (e.g. PPI plus sucralfate).

Our study has limitations. First, given our emphasis on early prediction, we did not assess the impact of in-hospital procedures, complications, and medications (such as antiplatelets, anticoagulants, glucocorticoids, selective serotonin reuptake inhibitors [30]), which may influence the risk of GIB. Second, since we only have information on new-onset GIB during hospitalization without documentation of the exact date, it remains unclear whether patients with a longer length of stay are more likely to develop GIB or if diagnosis of GIB leads to a longer hospitalization. Third, the generalizability of our study may be limited since the datasets comprised adults with ischemic stroke admitted to hospitals, and we excluded outpatients and those with TIA and hemorrhagic stroke. Fourth, the source of GIB and the clinical severity of GIB were not determined. Fifth, the information on pre-existing comorbidities was based on patients self-report and verified by chart review. Even though, we cannot sure for collecting all information on pre-existing comorbidities, especially a history of GIB. Finally, the AIS-GIB score needed to be further validated in different populations.

## Conclusion

We found that the AIS-GIB score is a valid clinical grading scale to predict the risk of in-hospital GIB after AIS. Further studies on the effect of the AIS-GIB score on reducing GIB and improving outcome after AIS are warranted.

## Key messages

1. Age, gender, certain pre-existing conditions (hypertension, hepatic cirrhosis, peptic ulcer disease, previous GIB), pre-stroke dependence, admission NIHSS score, GCS score, and OCSP subtype proved to be independent predictors for in-hospital GIB,based on which an 18-point GIB-AIS was developed.

2. The GIB-AIS showed good discrimination and calibration in large derivation, internal and external validation cohorts.

3. By sensitive analysis, the AIS-GIB demonstrated to be valid and significant for patients with different age, gender, and length of hospital stay.

4. Further validation of the GIB-AIS in different populations is needed.

## Abbreviations

GIB: Gastrointestinal bleeding; H_2_RA: Histamine H_2_ receptor antagonist; PPI: Proton pump inhibitor; AIS-GIB score: Acute Ischemic Stroke associated Gastrointestinal Bleeding Score; CNSR: China National Stroke Registry; CICAS: the Chinese Intracranial Atherosclerosis Study; AIS: Acute ischemic stroke; TIA: Transient ischemic attack; WHO: World Health Organization; CT: Computerized tomography; MRI: Magnetic resonance imaging; mRS: modified Rankin Scale; COPD: Chronic obstructive pulmonary disease; NIHSS: National Institutes of Health Stroke Scale score; GCS: Glasgow Coma scale; OCSP: Oxfordshire Community Stroke Project; PACI: Partial anterior circulation infarct; TACI: Total anterior circulation infarct; LACI: Lacunar infarction; POCI: Posterior circulation infarct; t-PA: Tissue plasminogen activator; VIF: Variance inflation factor; AUROC: Area under the receiver operating characteristic curve; SBP: Systolic blood pressure; DBP: Diastolic blood pressure; IQR: Interquartile range; OR: Odds ratio; C.I: Confidence interval.

## Competing interests

The authors declare that they have no competing interests.

## Authors’ contributions

RJ, ABS, and YW (Yongjun Wang) conceived of the study, participated in its design, and drafted the manuscript. HS and RJ carried out statistical analysis. RJ, HS, YP, PW, GL, YW (Yilong Wang), HL, XZ, ABS, and YW (Yongjun Wang) participated in analysis or interpretation of data, and revised the manuscript for important intellectual content. All authors read and approved the final manuscript.

## Pre-publication history

The pre-publication history for this paper can be accessed here:

http://www.biomedcentral.com/1471-230X/14/130/prepub

## Supplementary Material

Additional file 1: Table S1 Univariate analysis: predictors of GIB after AIS in the derivation cohort (n = 8,820). Showed univariate predictor of GIB after AIS in the derivation cohort. **Figure S1.** Plot of observed versus predicted risk of in-hospital GIB after AIS in the derivation, internal and external validation cohorts. Showed plot of observed versus predicted risk of GIB with 95% confidence intervals in the derivation and validation cohorts according to 10 deciles of predicted risk. Overall, there was a very high correlation between observed and predicted risk in the derivation cohort **(A)** (n = 8,820; r = 0.99, P < 0.001), internal validation cohort **(B)** (n = 5,882; r = 0.96, P < 0.001) and external validation cohort **(C)** (n = 2,938, r = 0.91, P < 0.001), which indicated excellent calibration. **Appendix A.** The CNSR and CICAS investigators. **Appendix B.** Institutional review board within the CNSR and CICAS network. Click here for file

## References

[B1] Wijdicks EF Fulgham JR Batts KP Gastrointestinal bleeding in stroke Stroke 1994 25 11 2146 2148 7974536 10.1161/01.str.25.11.2146

[B2] Davenport RJ Dennis MS Warlow CP Gastrointestinal hemorrhage after acute stroke Stroke 1996 27 3 421 424 8610306 10.1161/01.str.27.3.421

[B3] Johnston KC Li JY Lyden PD Hanson SK Feasby TE Adams RJ Faught RE Jr Haley EC Jr Medical and neurological complications of ischemic stroke: experience from the RANTTAS trial. RANTTAS Investigators Stroke 1998 29 2 447 453 9472888 10.1161/01.str.29.2.447

[B4] Roth EJ Lovell L Harvey RL Heinemann AW Semik P Diaz S Incidence of and risk factors for medical complications during stroke rehabilitation Stroke 2001 32 2 523 529 11157192 10.1161/01.str.32.2.523

[B5] Misra UK Kalita J Pandey S Mandal SK Predictors of gastrointestinal bleeding in acute intracerebral haemorrhage J Neurol Sci 2003 208 1–2 25 29 12639721 10.1016/s0022-510x(02)00415-x

[B6] O'Donnell MJ Kapral MK Fang J Saposnik G Eikelboom JW Oczkowski W Silva J Gould L D'Uva C Silver FL Investigators of the Registry of the Canadian Stroke Network Gastrointestinal bleeding after acute ischemic stroke Neurology 2008 71 9 650 655 18685137 10.1212/01.wnl.0000319689.48946.25

[B7] Hsu HL Lin YH Huang YC Weng HH Lee M Huang WY Lee JD Gastrointestinal hemorrhage after acute ischemic stroke and its risk factors in Asians Eur Neurol 2009 62 4 212 218 19622888 10.1159/000229018

[B8] Chen CM Hsu HC Chuang YW Chang CH Lin CH Hong CZ Study on factors affecting the occurrence of upper gastrointestinal bleeding in elderly acute stroke patients undergoing rehabilitation J Nutr 2011 15 8 632 636 10.1007/s12603-011-0052-2 PMC12878982 21968857

[B9] Hamidon BB Raymond AA The risk factors of gastrointestinal bleeding in acute ischaemic stroke Med J Malaysia 2006 61 3 288 291 17240577

[B10] Lai KC Lam SK Chu KM Wong BC Hui WM Hu WH Lau GK Wong WM Yuen MF Chan AO Lai CL Wong J Lansoprazole for the prevention of recurrences of ulcer complications from long-term low-dose aspirin use N Engl J Med 2002 346 26 2033 2038 12087138 10.1056/NEJMoa012877

[B11] Bhatt DL Scheiman J Abraham NS Antman EM Chan FK Furberg CD Johnson DA Mahaffey KW Quigley EM American College of Cardiology Foundation Task Force on Clinical Expert Consensus D ACCF/ACG/AHA 2008 expert consensus document on reducing the gastrointestinal risks of antiplatelet therapy and NSAID use: a report of the American College of Cardiology Foundation Task Force on Clinical Expert Consensus Documents Circulation 2008 118 18 1894 1909 18836135 10.1161/CIRCULATIONAHA.108.191087

[B12] Ng FH Wong SY Lam KF Chang CM Lau YK Chu WM Wong BC Gastrointestinal bleeding in patients receiving a combination of aspirin, clopidogrel, and enoxaparin in acute coronary syndrome Am J Gastroenterol 2008 103 4 865 871 18177451 10.1111/j.1572-0241.2007.01715.x

[B13] Abraham NS Hlatky MA Antman EM Bhatt DL Bjorkman DJ Clark CB Furberg CD Johnson DA Kahi CJ Laine L Mahaffey KW Quigley EM Scheiman J Sperling LS Tomaselli GF ACCF/ACG/AHA 2010 Expert Consensus Document on the concomitant use of proton pump inhibitors and thienopyridines: a focused update of the ACCF/ACG/AHA 2008 expert consensus document on reducing the gastrointestinal risks of antiplatelet therapy and NSAID use: a report of the American College of Cardiology Foundation Task Force on Expert Consensus Documents Circulation 2010 122 24 2619 2633 21060077 10.1161/CIR.0b013e318202f701

[B14] Bhatt DL Cryer BL Contant CF Cohen M Lanas A Schnitzer TJ Shook TL Lapuerta P Goldsmith MA Laine L Scirica BM Murphy SA Cannon CP COGENT Investigators Clopidogrel with or without omeprazole in coronary artery disease N Engl J Med 2010 363 20 1909 1917 20925534 10.1056/NEJMoa1007964

[B15] Earnshaw SR Scheiman J Fendrick AM McDade C Pignone M Cost-utility of aspirin and proton pump inhibitors for primary prevention Arch Intern Med 2011 171 3 218 225 21325111 10.1001/archinternmed.2010.525 PMC3137269

[B16] Schirmer CM Kornbluth J Heilman CB Bhardwaj A Gastrointestinal prophylaxis in neurocritical care Neurocrit Care 2012 16 1 184 193 21748505 10.1007/s12028-011-9580-1

[B17] Lin PC Chang CH Hsu PI Tseng PL Huang YB The efficacy and safety of proton pump inhibitors vs histamine-2 receptor antagonists for stress ulcer bleeding prophylaxis among critical care patients: a meta-analysis Crit Care Med 2010 38 4 1197 1205 20173630 10.1097/CCM.0b013e3181d69ccf

[B18] Alhazzani W Alshahrani M Moayyedi P Jaeschke R Stress ulcer prophylaxis in critically ill patients: review of the evidence Polskie Archiwum Medycyny Wewnetrznej 2012 122 3 107 114 22354363 10.20452/pamw.1173

[B19] Wang Y Cui L Ji X Dong Q Zeng J Wang Y Zhou Y Zhao X Wang C Liu L Nguyen-Huynh MN Claiborne Johnston S Wong L Li H China National Stroke Registry Investigators The China National Stroke Registry for patients with acute cerebrovascular events: design, rationale, and baseline patient characteristics Int J Stroke 2011 6 4 355 361 21609414 10.1111/j.1747-4949.2011.00584.x

[B20] Stroke--1989 Recommendations on stroke prevention, diagnosis, and therapy. Report of the WHO Task Force on Stroke and other Cerebrovascular Disorders Stroke 1989 20 10 1407 1431 2799873 10.1161/01.str.20.10.1407

[B21] Wang YL Wong Y Soo Y Pu Y Wong KL the Chinese IntraCranial AtheroSclerosis (CICAS) Study Group A Multicenter Study of the Prevalence and Outcomes of Intracranial Large Artery Atherosclerosis among Stroke and TIA Patients in China Stroke 2012 43 A 120

[B22] Bamford J Sandercock P Dennis M Burn J Warlow C Classification and natural history of clinically identifiable subtypes of cerebral infarction Lancet 1991 337 8756 1521 1526 1675378 10.1016/0140-6736(91)93206-o

[B23] Sullivan LM Massaro JM D'Agostino RB Sr Presentation of multivariate data for clinical use: The Framingham Study risk score functions Stat Med 2004 23 10 1631 1660 15122742 10.1002/sim.1742

[B24] Cook NR Use and misuse of the receiver operating characteristic curve in risk prediction Circulation 2007 115 7 928 935 17309939 10.1161/CIRCULATIONAHA.106.672402

[B25] Moscucci M Fox KA Cannon CP Klein W Lopez-Sendon J Montalescot G White K Goldberg RJ Predictors of major bleeding in acute coronary syndromes: the Global Registry of Acute Coronary Events (GRACE) Eur Heart J 2003 24 20 1815 1823 14563340 10.1016/s0195-668x(03)00485-8

[B26] Mangi AA Christison-Lagay ER Torchiana DF Warshaw AL Berger DL Gastrointestinal complications in patients undergoing heart operation: an analysis of 8709 consecutive cardiac surgical patients Ann Surg 2005 241 6 895 901 discussion 901-894 15912039 10.1097/01.sla.0000164173.05762.32 PMC1357169

[B27] Moukarbel GV Signorovitch JE Pfeffer MA McMurray JJ White HD Maggioni AP Velazquez EJ Califf RM Scheiman JM Solomon SD Gastrointestinal bleeding in high risk survivors of myocardial infarction: the VALIANT Trial Eur Heart J 2009 30 18 2226 2232 19556260 10.1093/eurheartj/ehp256

[B28] Chan KH Mann KS Lai EC Ngan J Tuen H Yue CP Factors influencing the development of gastrointestinal complications after neurosurgery: results of multivariate analysis Neurosurgery 1989 25 3 378 382 2771008 10.1097/00006123-198909000-00010

[B29] Schaller BJ Graf R Jacobs AH Pathophysiological changes of the gastrointestinal tract in ischemic stroke Am J Gastroenterol 2006 101 7 1655 1665 16863574 10.1111/j.1572-0241.2006.00540.x

[B30] Castro VM Gallagher PJ Clements CC Murphy SN Gainer VS Fava M Weilburg JB Churchill SE Kohane IS Iosifescu DV Smoller JW Perlis RH Incident user cohort study of risk for gastrointestinal bleed and stroke in individuals with major depressive disorder treated with antidepressants BMJ Open 2012 2 2 e000544 10.1136/bmjopen-2011-000544 PMC3330255 22466034

