# Supplementary material for: Risk score to predict gastrointestinal bleeding after acute ischemic stroke
Source: BMC Gastroenterol. 2014 Jul 25;14:130. doi: 10.1186/1471-230X-14-130 (PMC4120715; doi:10.1186/1471-230X-14-130)
Supplement: Additional file 1: Table S1 — Univariate analysis: predictors of GIB after AIS in the derivation cohort (n = 8,820). Showed univariate predictor of GIB after AIS in the derivation cohort. Figure S1. Plot of observed versus predicted risk of in-hospital GIB after AIS in the derivation, internal and external validation cohorts. Showed plot of observed versus predicted risk of GIB with 95% confidence intervals in the derivation and validation cohorts according to 10 deciles of predicted risk. Overall, there was a very high correlation between observed and predicted risk in the derivation cohort (A) (n = 8,820; r = 0.99, P < 0.001), internal validation cohort (B) (n = 5,882; r = 0.96, P < 0.001) and external validation cohort (C) (n = 2,938, r = 0.91, P < 0.001), which indicated excellent calibration. Appendix A. The CNSR and CICAS investigators. Appendix B. Institutional review board within the CNSR and CICAS network. [file 1471-230X-14-130-S1.doc]

# Supplementary materials

**Table S**1.Univariate analysis: predictors of GIB after AIS in the derivation cohort (n=8,820)

|  | Increment/categories | O.R. | 95% C.I. | P value |
| --- | --- | --- | --- | --- |
| Demographics |  |  |  |  |
| Age, y | 1 year increase | 1.03 | 1.02-1.04 | <0.001 |
| Gender | male vs. female | 1.11 | 1.02-1.32 | 0.03 |
| Risk factors |  |  |  |  |
| Hypertension | yes vs. no | 1.33 | 1.06-1.67 | 0.01 |
| Diabetes mellitus | yes vs. no | 1.02 | 0.79-1.31 | 0.88 |
| Hyperlipidemia | yes vs. no | 0.97 | 0.69-1.36 | 0.86 |
| Atrial fibrillation | yes vs. no | 1.72 | 1.24-2.39 | 0.001 |
| Coronary artery disease | yes vs. no | 1.43 | 1.09-1.87 | 0.009 |
| Peripheral artery disease | yes vs. no | 1.79 | 0.65-4.90 | 0.26 |
| History of stroke/TIA | yes vs. no | 1.72 | 1.39-2.12 | <0.001 |
| Smoking | yes vs. no | 1.02 | 0.82-1.26 | 0.87 |
| Heavy alcohol consumption | yes vs. no | 1.08 | 0.82-1.44 | 0.58 |
| Other coexistent conditions |  |  |  |  |
| Congestive heart failure | yes vs. no | 1.89 | 1.07-3.33 | 0.03 |
| Valvular heart disease | yes vs. no | 1.04 | 0.53-2.04 | 0.91 |
| COPD | yes vs. no | 1.81 | 0.84-3.90 | 0.13 |
| Hepatic cirrhosis | yes vs. no | 6.59 | 2.95-14.8 | <0.001 |
| Peptic ulcer or previous GI bleed | yes vs. no | 3.59 | 2.52-5.11 | <0.001 |
| Renal failure | yes vs. no | 3.99 | 0.51-31.2 | 0.19 |
| Arthritis | yes vs. no | 1.01 | 0.55-1.86 | 0.97 |
| Dementia | yes vs. no | 2.44 | 1.32-4.53 | 0.005 |
| Cancer | yes vs. no | 1.62 | 0.85-3.07 | 0.14 |
| Pre-stroke disability (mRS>3) | mRS>3 vs. mRS<2 | 3.29 | 2.57-4.22 | <0.001 |
| Pre-admission antiplatelet therapy | yes vs. no | 1.20 | 0.92-1.57 | 0.18 |
| Pre-admission anticoagulation therapy | yes vs. no | 1.24 | 0.65-2.34 | 0.51 |
| Admission NIHSS | per 1 increase | 1.10 | 1.09-1.11 | <0.001 |
| Admission GCS, median | per 1 decrease | 0.78 | 0.76-0.80 | <0.001 |
| Admission systolic blood pressure | per 10 mmHg increase | 1.02 | 0.97-1.07 | 0.43 |
| Admission diastolic blood pressure | per 10 mmHg increase | 1.01 | 0.93-1.10 | 0.78 |
| OCSP subtypes |  |  |  |  |
| Lacunar infarction (LACI) |  | 1.00 | … | … |
| Partial anterior circulation infarct (PACI) | PACI vs. LACI | 0.99 | 0.72-1.37 | 0.97 |
| Total anterior circulation infarct (TACI) | TACI vs. LACI | 2.99 | 2.07-4.33 | <0.001 |
| Posterior circulation infarct (POCI) | POCI vs. LACI | 2.13 | 1.50-3.01 | <0.001 |
| Intravenous t-PA within 3h after onset | yes vs. no | 1.21 | 0.45-3.28 | 0.71 |
| Antithrombotic therapy on admission | yes vs. no | 0.83 | 0.63-1.08 | 0.18 |
| Anticoagulation therapy on admission | yes vs. no | 1.23 | 0.65-2.34 | 0.51 |
| Length of hospital stay | per 1 day increase | 1.02 | 1.02-1.03 | <0.001 |

Abbreviation: C.I., confidence interval; COPD, chronic obstructive pulmonary disease; mRS, modified Rankin Scale; NIHSS, National Institutes of Health Stroke Scale score; GCS, Glasgow Coma Scale; OCSP, Oxfordshire Community Stroke Project; t-PA, tissue plasminogen activator

| 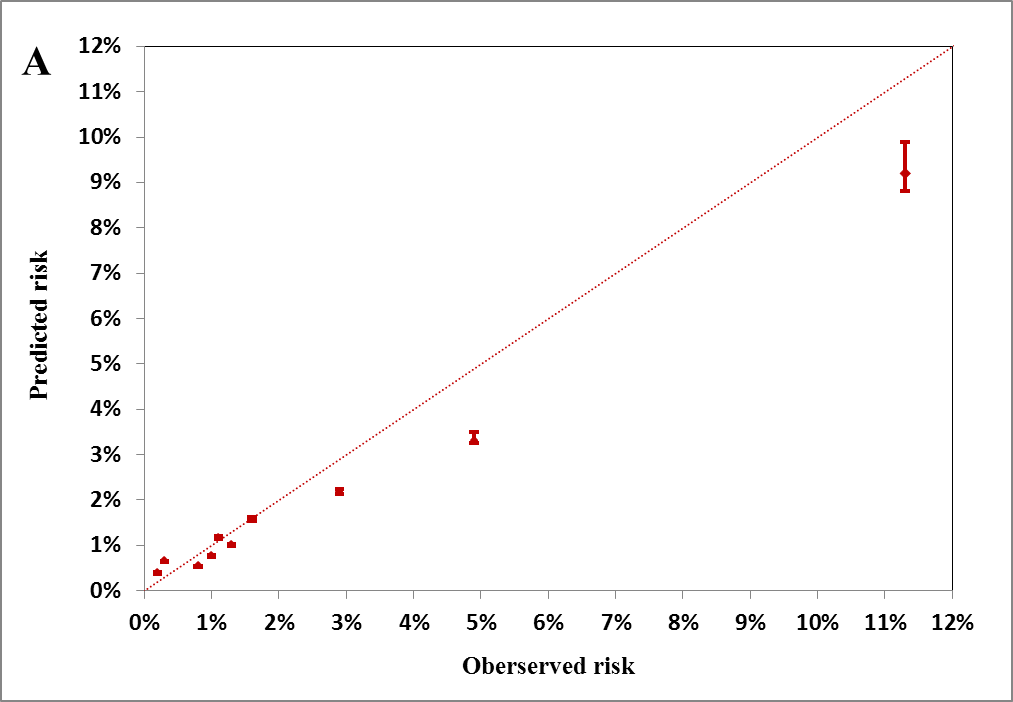 |
| --- |
| 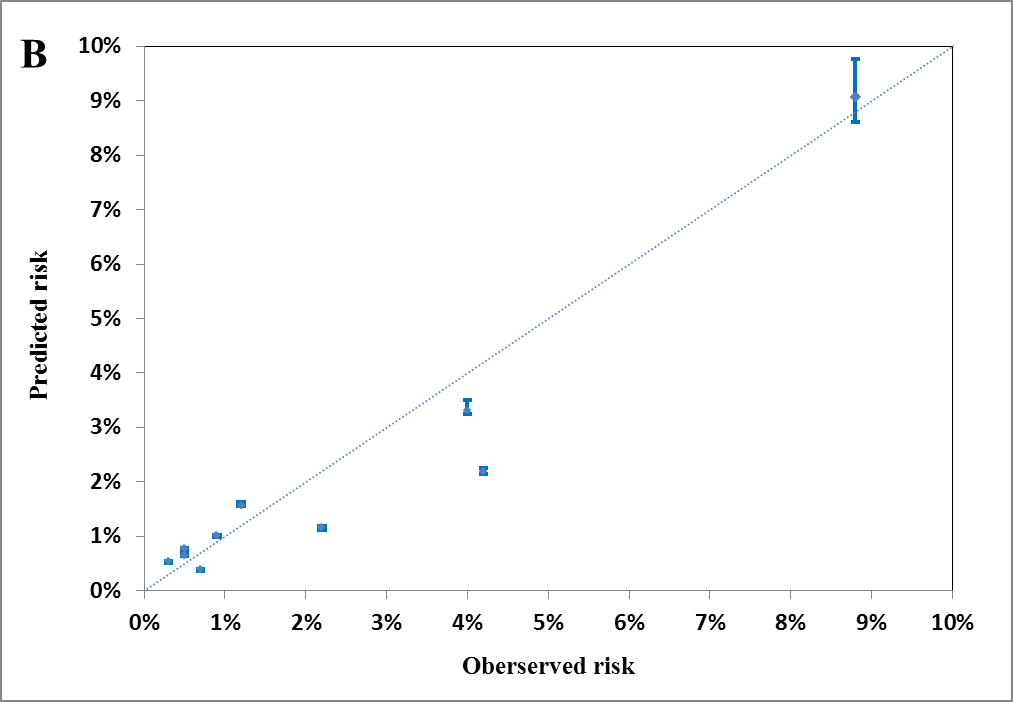 |
| 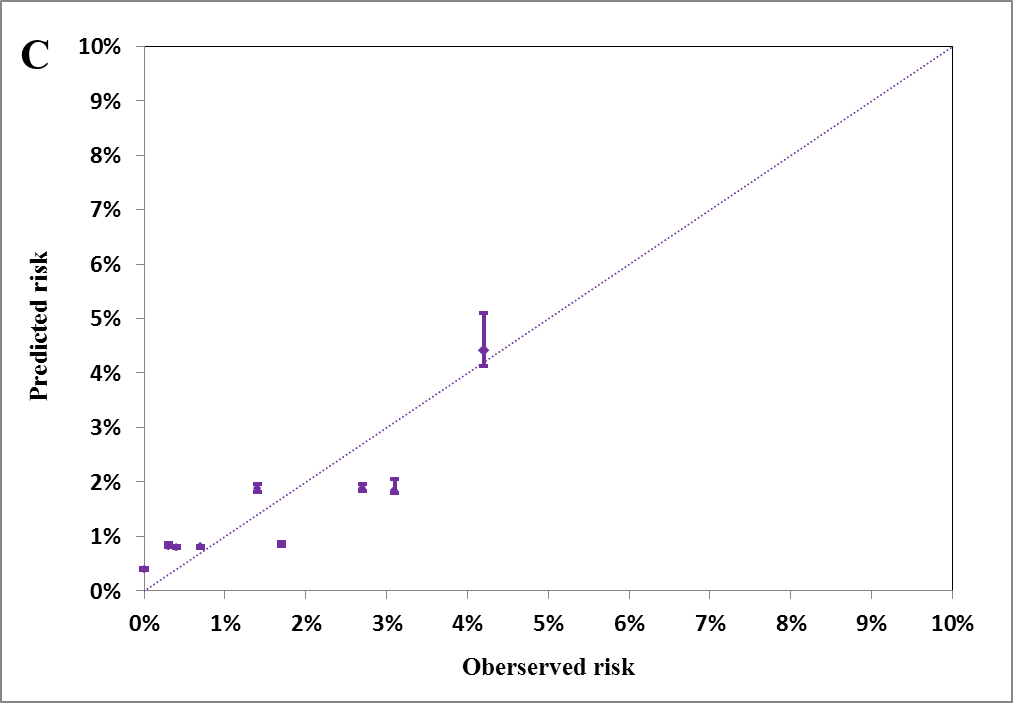 |
| Figure S1. Plot of observed versus predicted risk of in-hospital GIB after AIS in the derivation, internal and external validation cohorts |

**Figure legend**

Plot of observed versus predicted risk of GIB with 95% confidence intervals in the derivation and validation cohorts according to 10 deciles of predicted risk. Overall, there was a very high correlation between observed and predicted risk in the derivation cohort (A) (n=8,820; r=0.99, P<0.001), internal validation cohort (B) (n=5,882; r=0.96, P<0.001) and external validation cohort (C) (n=2,938, r=0.91, P<0.001), which indicated excellent calibration.

Appendix A: The CNSR and CICAS investigators

Yongjun Wang, Beijing Tiantan Hospital; Qi Bi, Beijing Anzhen Hospital; Weiwei Zhang, Beijing Military District Gengral hospital of Chinese People’s Liberation Army; Liying Cui, Peking Union Medical College Hospital of Peking University; Yuheng Sun, Beijing Jishuitan Hospital; Maolin He, Beijing Shijitan Hospital; Dongsheng Fan, Peking University Third Hospital; Xunming Ji, Beijing Xuanwu Hospital; Jimei Li, Beijing Friendship Hospital Affiliated to Capital Medical University; Fang Zhang, Beijing Guangwai Hospital; Kai Feng, Beijing Shunyi District Hospital; Xiaojun Zhang, Beijing Tongren Hospital; Yansheng Li, Shanghai Renji Hospital; Shaoshi Wang, Shanghai First Municipal People’s Branch hospital; Wei Fan, Zhongshan Hospital of Fudan University; Zhenguo Liu, Xin Hua Hospital Affiliated to Shanghai Jiao Tong University; Xiaojiang Sun, The sixth People’sHospital Affiliated to Shanghai JiaoTong University; Wei Li, Shanghai Ninth People’s Hospital Affiliated to Shanghai JiaoTong University; Jianrong Liu, ShanghaiRuijin Hospital; Xu Chen, Shanghai 8th People’s Hospital; Qingke Bai, Pudong New Area People’s Hospital; Dexiang Gu, Shanghai Yangpu Area Shidong Hospital; Xin Li, Shanghai Yangpu Area Center Hospital; Qiang Dong, Huashan Hospital of Fudan University; Yan Cheng, Tianjin Medical University Gengeal Hospital; Lan Yu, Tianjin Huanhu Hospital; Bin Li, Dagang Oilfield Gengeal Hospital; Tongyu Wang, Bohai Oilfield Hospital; Kun Zhao, Baodi District People’s Hospital of Tianjin; Chaodong Zhang, The First Affiliated Hospital of China Medical University; Dingbo Tao, The First Afflicated Hospital of Dlian Medical University; Lin Yin, The Second Affiliated Hospital of Dlian Medical University; Fang Qu, Dlian Second People’s hospital; Jingbo Zhang, Dlian Third People’s hospital; Jianfeng Wang, Dalian Central hospital; Ying Lian, Dalian Economic and Technological Development District Hospital; Fang Qu, Shenying Military District General hospital of Chinese People’s Liberation Army; Jun Fan, Shenyang Military District 202 Hospital; Ying Gao, National Traditional Chinese Medicine (TCM)Thrombus Treatment Center of Liaoning Province; Mingdong Cheng, En’liang hopital of Tai’an County; Jiang Wu, The First Clinical College of Jilin University; Huashan Sun, Jilin Chemical Industrial Group General hopital; Jinying Li, Jilin Oilfield General Hospital; Guozhong Li, The First Clinical College of Harbin Medical University; Yulan Zhu, The Second Clinical College of Harbin Medical University; Zichao Yang, The Fourth Clinical College of Harbin Medical University; Fengmin Yang, Daqing Oilfield General Hospital; Jun Zhou, Mudan Jiang Second hospital of Hailongjiang Province; Minxia Guo, Shaanxi Provincial People’s Hospital; Zhengyi Li, The First Afflicated Hospital of Medical College of Xian Jiaotong University; Qilin Ma, The First Hospital of Xiamen; Renbin Huang, Chenzhou First People’s Hospital; Bo Xiao, Xiangya Hospital of Centre-south University; Kangning Chen, Southwest Hospital; Xinyue Qin, The First Affiliated Hospital of Chongqing Medical University; Changlin Hu, The Second Affiliated Hospital of Chongqing Medical University; Li Gao, Chengdu Third Municipal People’s Hospital; Jinsheng Zeng, The First Affiliated Hospital of Sun Yat-Sen University; Anding Xu, The First Affiliated Hospital of Jinan University; Xiong Zhang, Guangdong People’s Hospital; Ming Shao, The First Affiliated Hospital of Guangzhou Medical University; Feng Qi, LiWan Hospital of GuangZhou Medical College; Weimin Xiao, Dungun Municipal People’s Hospital; Suping Zhang, Guangzhou Red Cross Hospital; Xiaoping Pan, Guangzhou First TMUNICIPAL People’s Hospital; Suyue Pan, Nan Fang Hospital; Yefeng Cai, Guangdong Provincial Hospital of Traditional Chinese Medicine; Qi Wan, Jiang Su People’s Hospital; Yun Xu, Drum Tower Hospital Affiliated to Nanjing Medical University Upper First-class Hospital; KaiFu Ke, he Affiliated Hospital of Nantong University Upper First class Hospital; Yuenan Kong,Wuxi Second People’s Hospital Upper First-class Hospital; Qing Di, Neurology Hospital Affiliated to Nanjing Medical University Upper First-class Hospital; Fengyang Shao, Jiangsu Province Lianyungang Hospital of TCM Upper First-class Hospital; Yajun Jiang, Jiangsu Province Hospital of TCM Upper First-class Hospital; Daming Wang, The First People’s Hospital of Changzhou Upper First-class Hospital; Li Guo, The Second Hospital of Hebei Medical University; Wencui Xue, Qinhuangdao C.

Appendix B: Institutional review board within the CNSR and CICAS network

Institutional review board at Beijing Tiantan Hospital; Institutional review board at Beijing Anzhen Hospital; Institutional review board at Beijing Military District Gengral hospital of Chinese People’s Liberation Army; Institutional review board at Peking Union Medical College Hospital of Peking University; Institutional review board at Beijing Jishuitan Hospital; Institutional review board at Beijing Shijitan Hospital; Institutional review board at Peking University Third Hospital; Institutional review board at Beijing Xuanwu Hospital; Institutional review board at Beijing Friendship Hospital Affiliated to Capital Medical University; Institutional review board at Beijing Guangwai Hospital; Institutional review board at Beijing Shunyi District Hospital; Institutional review board at Beijing Tongren Hospital; Institutional review board at Shanghai Renji Hospital; Institutional review board at Shanghai First Municipal People’s Branch hospital; Institutional review board at Zhongshan Hospital of Fudan University; Institutional review board at Xin Hua Hospital Affiliated to Shanghai Jiao Tong University; Institutional review board at the sixth People’sHospital Affiliated to Shanghai JiaoTong University; Institutional review board at Shanghai Ninth People’s Hospital Affiliated to Shanghai JiaoTong University; Institutional review board at ShanghaiRuijin Hospital; Institutional review board at Shanghai 8th People’s Hospital; Institutional review board at Pudong New Area People’s Hospital; Institutional review board at Shanghai Yangpu Area Shidong Hospital; Institutional review board at Shanghai Yangpu Area Center Hospital; Institutional review board at Huashan Hospital of Fudan University; Institutional review board at Tianjin Medical University Gengeal Hospital; Institutional review board at Tianjin Huanhu Hospital; Institutional review board at Dagang Oilfield Gengeal Hospital; Institutional review board at Bohai Oilfield Hospital; Institutional review board at Baodi District People’s Hospital of Tianjin; Institutional review board at The First Affiliated Hospital of China Medical University; Institutional review board at The First Afflicated Hospital of Dlian Medical University; Institutional review board at The Second Affiliated Hospital of Dlian Medical University; Institutional review board at Dlian Second People’s hospital; Institutional review board at Dlian Third People’s hospital; Institutional review board at Dalian Central hospital; Institutional review board at Dalian Economic and Technological Development District Hospital; Institutional review board at Shenying Military District General hospital of Chinese People’s Liberation Army; Institutional review board at Shenyang Military District 202 Hospital; Institutional review board at National Traditional Chinese Medicine (TCM) Thrombus Treatment Center of Liaoning Province; Institutional review board at En’liang hopital of Tai’an County; Institutional review board at The First Clinical College of Jilin University; Institutional review board at Jilin Chemical Industrial Group General hopital; Institutional review board at Jilin Oilfield General Hospital; Institutional review board at The First Clinical College of Harbin Medical University; Institutional review board at The Second Clinical College of Harbin Medical University; Institutional review board at The Fourth Clinical College of Harbin Medical University; Institutional review board at Daqing Oilfield General Hospital; Institutional review board at Mudan Jiang Second hospital of Hailongjiang Province; Institutional review board at Shaanxi Provincial People’s Hospital; Institutional review board at The First Afflicated Hospital of Medical College of Xian Jiaotong University; Institutional review board at The First Hospital of Xiamen; Institutional review board at Chenzhou First People’s Hospital; Institutional review board at Xiangya Hospital of Centre-south University; Institutional review board at Southwest Hospital; Institutional review board at The First Affiliated Hospital of Chongqing Medical University; Institutional review board at The Second Affiliated Hospital of Chongqing Medical University; Institutional review board at Chengdu Third Municipal People’s Hospital; Institutional review board at The First Affiliated Hospital of Sun Yat-Sen University; Institutional review board at The First Affiliated Hospital of Jinan University; Institutional review board at Guangdong People’s Hospital; Institutional review board at The First Affiliated Hospital of Guangzhou Medical University; Institutional review board at LiWan Hospital of GuangZhou Medical College; Institutional review board at Dungun Municipal People’s Hospital; Institutional review board at Guangzhou Red Cross Hospital; Institutional review board at Guangzhou First TMUNICIPAL People’s Hospital; Institutional review board at Nan Fang Hospital; Institutional review board at Guangdong Provincial Hospital of Traditional Chinese Medicine; Institutional review board at Jiang Su People’s Hospital; Institutional review board at Drum Tower Hospital Affiliated to Nanjing Medical University Upper First-class Hospital; Institutional review board at the Affiliated Hospital of Nantong University Upper First class Hospital; Institutional review board at Wuxi Second People’s Hospital Upper First-class Hospital; Institutional review board at Neurology Hospital Affiliated to Nanjing Medical University Upper First-class Hospital; Institutional review board at Jiangsu Province Lianyungang Hospital of TCM Upper First-class Hospital; Institutional review board at Jiangsu Province Hospital of TCM Upper First-class Hospital; Institutional review board at The First People’s Hospital of Changzhou Upper First-class Hospital; Institutional review board at The Second Hospital of Hebei Medical University; Institutional review board at Qinhuangdao C hospital.
